# 1225. *In Vitro* Activity of Aztreonam-Avibactam and Comparator Agents Against Enterobacterales Collected from Geriatric Patients in ICU and non-ICU wards, ATLAS Surveillance Program 2016-2019

**DOI:** 10.1093/ofid/ofab466.1417

**Published:** 2021-12-04

**Authors:** Sibylle Lob, Krystyna Kazmierczak, Francis Arhin, Daniel F Sahm

**Affiliations:** 1 IHMA, Inc., Schaumburg, IL; 2 Pfizer Canada, Kirkland, Quebec, Canada

## Abstract

**Background:**

Elevated resistance rates have been reported in ICUs. Aztreonam (ATM) combined with avibactam (AVI) is being developed for use against drug-resistant Enterobacterales (Ebact), including metallo-β-lactamase (MBL)-positive isolates. We examined the activity of ATM-AVI and comparators against Ebact isolates collected from geriatric patients in ICU and non-ICU wards as part of the ATLAS surveillance program.

**Methods:**

23754 non-duplicate Ebact isolates were collected in 53 countries in Asia/Pacific (excluding mainland China and India), Europe, Latin America, and Middle East/Africa from patients ≥65 years with lower respiratory tract (LRTI), urinary tract (UTI), skin and soft tissue (SSTI), intra-abdominal (IAI), and bloodstream (BSI) infections. Susceptibility testing was performed by CLSI broth microdilution and values interpreted using CLSI 2021 breakpoints. PCR and sequencing were used to determine the β-lactamase genes present in isolates with meropenem MIC >1 µg/mL, and *Escherichia coli*, *Klebsiella* spp. and *Proteus mirabilis* with ATM or ceftazidime MIC >1 µg/mL.

**Results:**

Susceptibility of the studied comparator agents was generally slightly lower among Ebact from BSI than other infection types (Table). Susceptibility was also generally lower among Ebact from ICU than non-ICU wards by up to 10 percentage points, and MIC_90_ values were up to 32-fold higher. ATM-AVI MIC_90_ values were within one doubling-dilution across all studied strata (0.12-0.25 µg/mL), were comparable to or lower than for meropenem in all strata, and were 2 to ≥9 dilutions lower than all other tested comparators. MBL-positive Ebact were found in 1.5% of LRTI (n=91), 1.2% of UTI (n=70), 1.1% of SSTI (n=52), 1.3% of BSI (n=49), and 0.7% of IAI isolates (n=22). MBL-positive rates were higher among ICU (1.7%, n=101) than non-ICU isolates (1.0%, n=183). ATM-AVI MIC_90_ values were 0.5 µg/mL against MBL-positive isolates from all ward and infection types except SSTI (MIC_90_ 0.25 µg/mL) and BSI (MIC_90_ 1 µg/mL), 2-4 dilutions lower than tigecycline and at least 5-10 dilutions lower than the other comparators.

Results Table

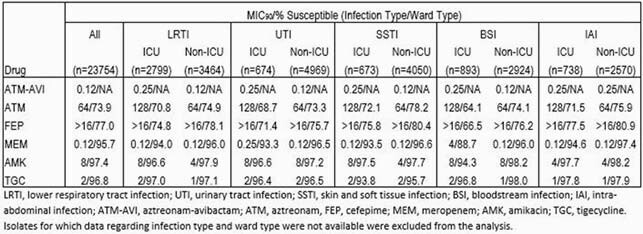

**Conclusion:**

ATM-AVI could provide a valuable therapeutic option for treatment of infections caused by Ebact in patients ≥65 years old in both ICU and non-ICU wards.

**Disclosures:**

**Sibylle Lob, PhD**, **IHMA** (Employee)**Pfizer, Inc.** (Independent Contractor) **Krystyna Kazmierczak, PhD**, **IHMA** (Employee)**Pfizer, Inc.** (Independent Contractor) **Francis Arhin, PhD**, **Pfizer, Inc.** (Employee) **Daniel F. Sahm, PhD**, **IHMA** (Employee)**Pfizer, Inc.** (Independent Contractor)

